# Different combinations of serotonin receptors regulate predatory and bacterial feeding behaviors in the nematode *Pristionchus pacificus*

**DOI:** 10.1093/g3journal/jkab011

**Published:** 2021-01-20

**Authors:** Yuuki Ishita, Takahiro Chihara, Misako Okumura

**Affiliations:** 1 Program of Biomedical Science, Graduate School of Integrated Sciences for Life, Hiroshima University, Higashi-Hiroshima, Hiroshima 739-8526, Japan; 2 Program of Basic Biology, Graduate School of Integrated Sciences for Life, Hiroshima University, Higashi-Hiroshima, Hiroshima 739-8526, Japan; 3 Department of Biological Science, Graduate School of Science, Hiroshima University, Higashi-Hiroshima, Hiroshima 739-8526, Japan

**Keywords:** feeding behavior, *Pristionchus pacificus*, serotonin receptor, predation

## Abstract

Feeding behavior is one of the most fundamental behaviors in animals, and regulation of this behavior is critical for proper food intake. The nematode *Pristionchus pacificus* exhibits dimorphism in feeding behavior, bacterial feeding and predatory feeding on other nematodes, and the latter behavior is assumed to be an evolutionarily novel behavior. Both types of feeding behavior are modulated by serotonin; however, the downstream mechanism that modulates these behaviors is still to be clarified. Here, we focused on serotonin receptors and examined their expression patterns in *P. pacificus*. We also generated knockout mutants of the serotonin receptors using the CRISPR/Cas9 system and examined feeding behaviors. We found that *Ppa-ser-5* mutants and the *Ppa-ser-1*; *Ppa-ser-7* double mutant decreased predation. Detailed observation of the pharyngeal movement revealed that the *Ppa-ser-1*; *Ppa-ser-7* double mutant reduces tooth movement, which is required for efficient predatory feeding. Conversely, *Ppa-ser-7* and *Ppa-mod-1* mutants decreased bacterial feeding. This study revealed that specific combinations of serotonin receptors are essential for the modulation of these distinct feeding behaviors, providing insight into the evolution of neural pathways to regulate novel feeding behavior.

## Introduction

Animals have evolved to change their diet to adapt to their surrounding environment. To achieve this, it is necessary to acquire new feeding behaviors by altering morphological structures such as feeding apparatus, physiological features such as composition of digestive juice, andbehavioral characteristics. Alteration in behavioral features should be accompanied by changes in the nervous system related to behaviors.

Nematodes have extremely diverged in feeding behavior (Yeates *et al.* 1993). To adapt to food sources such as bacteria, fungi, and plants, they have developed numerous types of feeding structures and regulatory systems of feeding behavior. The predatory nematode *Pristionchus pacificus* is an intriguing model to explore the mechanisms of feeding regulation and its evolution. *P. pacificus* belongs to the family Diplogastridae that has been established as a satellite model animal compared to *Caenorhabditis elegans* (Sommer *et al.* 1996). Various genetic tools such as annotated genome sequences (Dieterich *et al.* 2008; Rödelsperger *et al.* 2017), genome editing methods using the CRISPR/Cas9 system (Witte *et al.* 2015; Nakayama *et al.* 2020) and transformation of exogenous genes (Schlager *et al.* 2009; Namai and Sugimoto 2018) are available for *P. pacificus*. Moreover, neuronal connectomics in pharyngeal neurons and amphid sensory neurons have been identified (Bumbarger *et al.* 2013; Hong *et al.* 2019), increasing the potential for detailed behavioral analysis.

*P. pacificus* exhibits polyphenism in its feeding structure in response to the surrounding environment during development. One form is called the eurystomatous morph, which is characterized by a wider and shallow buccal cavity, a claw-like dorsal tooth, and several subventral teeth. The other is a stenostomatous morph, with a narrow, deep buccal cavity and a single flint-like tooth (Bento *et al.* 2010). *P. pacificus* with either mouth form feeds on bacterial food, while predatory feeding behavior is only seen in eurystomatous worms (Wilecki *et al.* 2015). While bacterial feeding is common in a broader range of taxonomic clades, predatory feeding behavior is observed in Diplogastridae nematodes (Blaxter *et al.* 1998). Thus, predatory feeding behavior is assumed to be an evolutionarily novel behavior and provides a suitable model for understanding the neural evolution of feeding behaviors.

The predatory feeding behavior in *P. pacificus* is characterized by the coordinated movement of pharyngeal pumping and tooth movement, enabling the efficient killing of prey (Wilecki *et al.* 2015; Okumura *et al.* 2017). A neuromodulator, serotonin, is a key molecule that regulates this behavior (Wilecki *et al.* 2015). Mutants of two enzymes essential for serotonin synthesis, *Ppa-tph-1*, and *Ppa-bas-1*, fail to induce efficient feeding on other nematodes. These mutants decrease tooth movement during feeding on other nematodes, disrupting coordinated feeding rhythms (Okumura *et al.* 2017). In contrast, exogenous serotonin solely induces the coordinated movement of pharyngeal pumping and tooth movement (Wilecki *et al.* 2015), suggesting that serotonin plays an essential role in the regulation of predatory feeding behavior. Serotonin also modulates the bacterial feeding rate in *P. pacificus* (Okumura *et al.* 2017). Compared with pharyngeal movements during predation, pharyngeal movement during bacterial feeding lacks tooth movements, and the pumping rate is faster (Wilecki *et al.* 2015). However, how serotonin modulates these different feeding modes and how the nervous systems evolve to modulate predatory feeding behavior remains unclear.

In this study, we present serotonin receptors that regulate predatory and bacterial feeding behaviors in *P. pacificus*. In the model nematode *C. elegans*, five serotonin receptors play a role in feeding behavior (Olde and McCombie 1997; Ranganathan *et al.* 2000; Hamdan *et al.*^1999^; Hobson *et al.* 2003; Tsalik *et al.* 2003; Hapiak *et al.* 2009; Ishita *et al.* 2020) . We examined the functions and expressions of all five serotonin receptors in *P. pacificus*. We found serotonin receptors were expressed in some orthologous cells between *C. elegans* and *P. pacificus*, while some differences in the expression patterns were observed between these species. We generated serotonin receptor mutants using CRISPR/Cas9 system and found that *Ppa-ser-1*, *Ppa-ser-7*, and *Ppa-ser-5* have significant roles in the regulation of predatory feeding behavior. We also determined that *Ppa-ser-7* and *Ppa-mod-1* play major roles in the modulation of bacterial feeding behavior. This study offers downstream genetic mechanisms of serotonin in the regulation of predatory and bacterial feeding behaviors in *P. pacificus* and insights into the evolution of a novel feeding behavior.

## Materials and methods

### Strains

All strains used in the experiments were maintained at 20° on standard NGM plates with *Escherichia coli* OP50 (Brenner 1974; Stiernagle 2006). The strains are listed in Supplementary Table S1.

### Molecular cloning

The *P. pacificus* orthologs of five *C. elegans* serotonin receptors were predicted as previously described (Baskaran *et al.* 2015). A 5' and 3' RACE was performed to determine the mRNA sequences of the serotonin receptors. The cDNA for RACE was prepared using the SMARTer^®^ RACE 5'/3' Kit (Clontech, 634858), and the target sequences were amplified using PrimeSTAR Max DNA polymerase I (TaKaRa, R045A) or KOD-Fx-Neo (TOYOBO, KFX-201). The amplicons were purified and sequenced using Sanger sequencing.

### Generation of transgenic animals

The promoter regions of the serotonin receptors were amplified using PrimeSTAR Max DNA polymerase I or KOD One PCR Master Mix (TOYOBO, KMM-101). The lengths of the promoter sequences were as follows: *Ppa-ser-1*p: 3.5 kb; *Ppa-ser-4*p: 4.5 kb; *Ppa-ser-5*p: 5.7 kb; *Ppa-ser-7*p: 7.1 kb; *Ppa-mod-1*p: 3.8 kb. The promoters for *Ppa-ser-4* and *Ppa-ser-7* include intergenic regions flanked by the end of predicted 3’ untranslated regions (UTRs) of upstream genes on the same strand and the start codon of genes of interest. For *Ppa-ser-1*p and *Ppa-ser-5*p, we used shorter sequences because of the difficulty of PCR amplification. *Ppa-mod-1*p has a longer sequence containing 151 bp of 3’ UTR of a predicted upstream gene due to the difficulty of plasmid construction. The primers used to amplify the promoters are shown in Supplementary Table S2. To examine the expression patterns of serotonin receptors, complex arrays were generated as previously described (Schlager *et al.* 2009). Transgenes and genomic DNA of PS312 were digested using appropriate digestion enzymes [*Ppa-egl-20p::Venus*, *Ppa-ser-4p::turboRFP*, *Ppa-ser-7p::turboRFP*, *Ppa-mod-1p::turboRFP*: FastDigest *Hin*dIII (Thermo Fischer, FD0504); *Ppa-ser-1p::turboRFP*: FastDigest *Hin*dIII and FastDigest *Bam*HI (Thermo Fischer, FD0054); *Ppa-ser-5p::turboRFP*: FastDigest *Kpn*I (Thermo Fischer, FD0524)]. The mixture containing *Ppa-egl-20p::Venus* (15 ng/µL) as an injection marker, the digested genomic DNA (60 ng/µL), and reporter genes for serotonin receptors (*Ppa-ser-1p::turboRFP*: 10 ng/µL; *Ppa-ser-4p::turboRFP*: 2 ng/µL; *Ppa-ser-5p::turboRFP*: 10 ng/µL; *Ppa-ser-7p::turboRFP*: 10–15 ng/µL; *Ppa-mod-1p::turboRFP*: 3 ng/µL) was injected in the gonads of young adults. The transgenic animals were screened using a fluorescent stereoscope (Leica).

The fluorescent images of the transgenic animals were obtained using an LSM microscope (Zeiss). Expressing cells were examined by manual scans of Z-stack images using ImageJ software (RRID: SCR_003070). The images in the head region of adult worms were analyzed for at least six animals for each mouth form per gene. The mouth form was evaluated as previously described (Serobyan *et al.* 2013).

### Quantification of serotonin receptor expression

To quantify the expression levels of serotonin receptors, quantitative reverse transcript PCR (qRT-PCR) was performed as previously described (Schuster and Sommer 2012). The wild-type PS312 strain was used as the eurystomatous-rich strain, and the *Ppa-eud-1* mutant was adopted as the all-stenostomatous strain (Ragsdale *et al.* 2013). A total of 100–120 young adult worms were manually selected, and the total RNA was extracted using an RNeasy Mini Kit (Qiagen, 74104). The cDNA was synthesized with a PrimeScript™ RT reagent Kit with gDNA Eraser (Perfect Real Time) (TaKaRa, RR047A) and qRT-PCR was performed with a CFX Connect Real-Time PCR Detection System (Bio-Rad, #1855201J1). PCR was performed using Brilliant III Ultra-Fast SYBR Green QPCR Master Mix (Agilent Technologies, 600882) as a DNA polymerase. Three technical replicates and three biological replicates were examined for each gene. We used *Ppa-cdc-42* gene expression as a reference gene (Schuster and Sommer 2012). Data were processed and plotted using CFX Manager 3.1 (RRID: SCR_017251). Primers for qRT-PCR are listed in Supplementary Table S3.

### Generation of serotonin receptor mutants using the CRISPR/Cas9 system

Serotonin receptor mutants were generated using the CRISPR/Cas9 genome editing system, as previously described (Witte *et al.* 2015). The guide RNA targets were selected using the CHOPCHOP web tool (https://chopchop.cbu.uib.no) (last accessed: 2021, Feb. 4th, RRID: SCR_015723). Target sequences for CRISPR/Cas9 mutagenesis are listed in Supplementary Table S4. The tracrRNA and target-specific crRNA (IDT), or synthesized single-guide RNAs (sgRNA) using the EnGen™ sgRNA Synthesis Kit, *S. pyogenes* (NEB #E3322S) were utilized as guide RNAs. In the latter case, single-stranded DNA oligos (Eurofins) were used as templates. A guide RNA and Cas9 protein (IDT) were mixed in a 2:1 molar ratio and incubated at 37° for 10 min to make a ribonucleoprotein (RNP) complex and diluted with nuclease-free water or TE buffer. The RNP complex was injected into the gonads of 1-day adult hermaphrodites. To generate *Ppa-ser-5 (cbh23)* and *Ppa-ser-1 (cbh58)* mutants, the *Ppa-prl-1* RNP complex was also injected as a co-injection marker (Nakayama *et al.* 2020). F0 worms were discarded 12–24 h after microinjection.

For mutation screening, the heteroduplex mobility assay (HMA) was performed as previously described (Nakayama *et al.* 2020). The primers for HMA are listed in Supplementary Table S5. The mutants were back-crossed at least three times with the original wild-type strain (PS312).

### Assays for feeding behaviors

Predatory behavioral assays were performed using previously established methods (Wilecki *et al.* 2015; Lightfoot *et al.* 2016; Okumura *et al.* 2017) with some modifications.

To perform the bite assay, *C. elegans* (N2) larvae were collected with M9 buffer and filtered by double 20-µm nylon filters. After concentrating *C. elegans* larvae via centrifugation, 2 µL of the larvae were placed on fresh NGM plates without *E. coli* food and the larvae were allowed to spread. One- to two-day-old *P. pacificus* was placed on the plates and allowed to recover for 30 min–1 h. Bite, kill, and feed events were counted for 10 min using a stereomicroscope. Each predatory event was determined as follows: bites, restriction of prey movement, kills, rupture of prey cuticles, and feeds, consuming prey body fluid (Figure 3A). The assay was performed blindly on at least two different days at 20°. In total, more than 15 animals per strain were examined.

The pumping rate was examined during predatory feeding, bacterial feeding, and exposure to exogenous serotonin. Thirty-five mm NGM plates were prepared for each experiment. The N2 larvae prepared as described above were placed on the plates for predatory feeding, and *E. coli* OP50 was seeded for bacterial pumping. One- to two-day-old *P. pacificus* adults were manually selected on the plates and allowed to recover for more than 30 min. To examine pharyngeal movements in response to exogenous serotonin, 30 µL of 10 mg/mL of serotonin (Sigma-Aldrich, H7752) in M9 buffer was applied onto the worms and a cover glass was loaded onto the ager plate. The pharyngeal movement was recorded for 15 s at 30 frames per second using a CCD camera with a DIC microscope (Leica). Tooth movement together with pharyngeal pumping during predation and exogenous serotonin exposure were recorded at 400× magnification, while bacterial pumping was recorded at 200× magnification. The video was manually scanned frame-by-frame using ImageJ software, and the numbers of pharyngeal pumping and tooth movements were recorded.

### Egg-laying assay

Egg-laying assays were performed using methods described previously (Okumura *et al.* 2017). Two- to three-day-old adult worms were loaded into 50 µL M9 buffer or 4 mg/mL serotonin solution in 96-well culture plates. After 2 h, the number of eggs was manually counted. The number of eggs in the uterus was counted in 3-day-old adult worms. The investigator was not blinded during this test.

### Locomotion assay

Paralyzing test for the *Ppa-ser-4* mutant was performed as previously described (Gürel *et al.* 2012). Two- to three-day-old adult worms were put into 50 µL M9 buffer with or without 30 mM serotonin in 96-well culture plates. After 1 h, the numbers of paralyzed worms and moving worms were counted. Worms bending the whole body smoothly were classified as the moving animals. Worms with movements only in the head region or with jerky movements in partial body parts were categorized into the paralyzed worms. For body bending assay, the number of body bending was examined in worms loaded into M9 buffer for 10 s. Worms that were not moving were excluded from this assay. The investigator was not blinded for this assay.

### Statistical analysis

The data were processed and analyzed using GraphPad Prism 7 (RRID: SCR_002798) and in the case of Figure 2H, Microsoft Excel (RRID: SCR_016137). Error bars represent the SEM, and all statistical tests used, and meanings of symbols can be found in the figure legends.

### Data availability

All animal strains shown in Supplementary Table S1 and plasmids used in this study are available upon request. The sequences of primers and guide RNA targets for CRISPR/Cas9 genome editing used in this study can be found in Supplementary Tables S2–S5. Supplementary Figures and Tables are available at figshare: https://doi.org/10.25387/g3.13413503.

## Results

### Gene structures and expression patterns of serotonin receptors in *P. pacificus*

**Figure 1:**
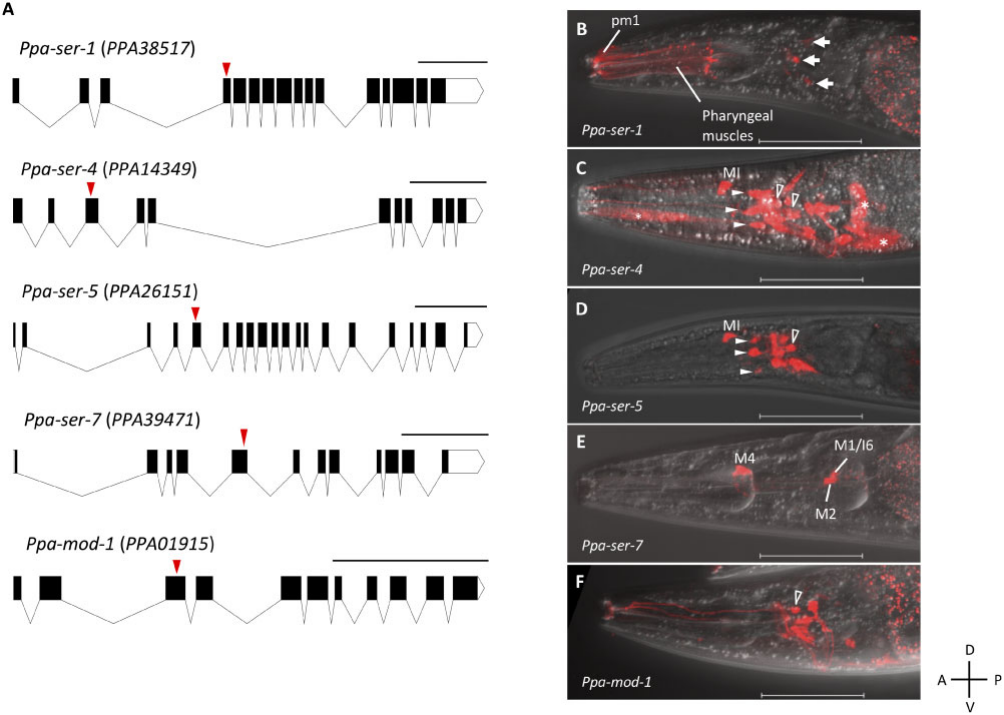
The gene structures and expression patterns of serotonin receptor orthologs in *P. pacificus.* (A) Gene structures of serotonin receptors. Black and white boxes represent exons and UTRs, respectively. Red arrowheads represent the positions of the targets of guide RNAs for CRISPR/Cas9-based knockout experiments. Scale bars, 1 kilobase pair. (B)–(F) Merged images of turboRFP fluorescence (shown in red) and DIC images in *Ppa-ser-1p::turboRFP* (B), *Ppa-ser-4p::turboRFP* (C), *Ppa-ser-5p::turboRFP* (D), *Ppa-ser-7p::turboRFP* (E), and *Ppa-mod-1p::turboRFP* (F) animals in the head region. All images were acquired from adult eurystomatous hermaphrodites. White arrows (B), white arrowheads (C and D), open arrowheads (C, D, and F), and asterisks (C) represent *Ppa-ser-1*-expressing head neurons, labial neurons, amphid neurons, and nonneuronal cells, respectively. A, anterior; P, posterior; D, dorsal; V, ventral. Scale bars, 50 µm.

To reveal the serotonergic modulation of feeding behaviors in *P. pacificus*, we first conducted molecular cloning of serotonin receptors. The orthologs of serotonin receptors were predicted using best-reciprocal hits, and coding sequences were determined by 5' and 3' RACE (Figure 1A). The following gene annotations were predicted as orthologs of serotonin receptors: *PPA38517* for *Ppa-ser-1*, *PPA14349* for *Ppa-ser-4*, *PPA26151* for *Ppa-ser-5*, *PPA39471* for *Ppa-ser-7*, and *PPA01915* for *Ppa-mod-1*. The amino acid sequences of serotonin receptors were highly conserved between *P. pacificus* and *C. elegans* (50.59% identity for SER-1, 60.71% for SER-4, 72.35% for SER-5, 51.86% for SER-7, 74.72% for MOD-1). We confirmed that there were no *Pristionchus*-specific paralogs of those serotonin receptors by BLASTP searches (data not shown). Thus, we considered those genes encoding orthologs of serotonin receptors identified in *C. elegans*.

We generated reporter lines containing the upstream region of each serotonin receptor gene and RFP to examine the expression patterns of these serotonin receptors in *P. pacificus* (Figure 1, B–F). *Ppa-ser-1* was expressed in the anterior pharyngeal muscles and several head neurons (Figure 1B, arrows). Notably, *Ppa-ser-1* expression was prominent in the pm1 pharyngeal muscle, where the dorsal tooth denticle attaches. *Ppa-ser-1* was weakly expressed in the anus (Supplementary Figure S1A). *Ppa-ser-4* was expressed in the MI pharyngeal neuron, and head neurons including several amphid neurons (Figure 1C, open arrowheads) and labial neurons (Figure 1C, white arrowheads). *Ppa-ser-4* was also expressed in nonneuronal cells in the head region (Figure 1C, asterisks) and several tail neurons (Supplementary Figure S1B). *Ppa-ser-5* was expressed in several head neurons including amphid neurons (Figure 1D, open arrowhead), labial neurons (Figure 1D, white arrowheads), and the MI pharyngeal neuron (Figure 1D). *Ppa-ser-4* and *Ppa-ser-5* seemed to be expressed redundantly in some of these cells. *Ppa-ser-5* was also expressed in the vulval muscles (Supplementary Figure S1C). *Ppa-ser-7* was expressed in some pharyngeal neurons, including M4, M1, M2, and I6 neurons, which were identified by their cell body positions and morphologies of neuronal processes (Figure 1E). *Ppa-ser-7* was also expressed in the vulval muscles, which are different from those expressing *Ppa-ser-5* (Supplementary Figure S1D). *Ppa-mod-1* was expressed in several head neurons, including a pair of amphid sensory neurons (Figure 1F, open arrowhead), but not expressed in any pharyngeal cells (Figure 1F). It was also expressed in gonadal cells, tail neurons, and slightly expressed in vulval neurons (Supplementary Figure S1, E–G). We compared the expression patterns and expression levels of serotonin receptors in eurystomatous and stenostomatous worms using microscopic observations and qRT-PCR. Although the expression level of all serotonin receptors tended to be higher in the *Ppa-eud-1* mutant, an all-stenostomatous strain (Supplementary Figure S2A), we could not find apparent differences in the expression patterns of serotonin receptors between the two mouth forms by microscopic observations (Supplementary Figure S2, B–F).

### Generation of serotonin receptor mutants using the CRISPR/Cas9 system

**Figure 2:**
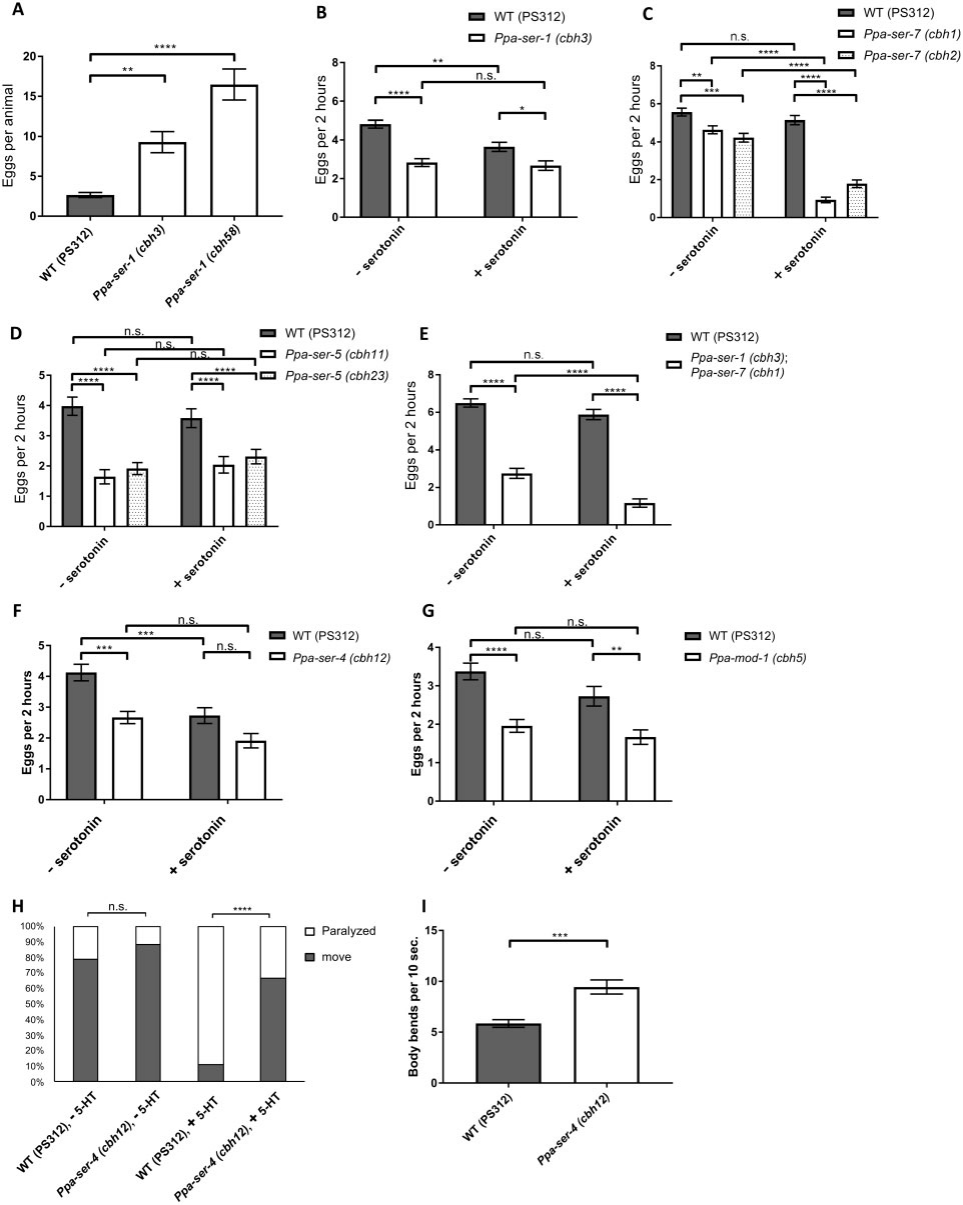
Phenotypic analysis of serotonin receptor mutants in egg-laying behavior and locomotion. (A) The number of eggs in the uterus of wild type and *Ppa-ser-1* mutants. Wild type, *n* = 48. *Ppa-ser-1 (cbh3)*, *n* = 47. *Ppa-ser-1 (cbh58)*, *n* = 46. Error bars represent the SEM. One-way ANOVA with Dunnett’s multiple comparison tests. **P < 0.01. ****P < 0.0001. (B)–(G) The number of eggs laid in M9 buffer and serotonin solution within 2 h in serotonin receptor mutants. (B) Wild type, *n* = 47. *Ppa-ser-1 (cbh3)*, *n* = 48. (C) Wild type, *n* = 143. *Ppa-ser-7 (cbh1)*, *n* = 96. *Ppa-ser-7 (cbh2)*, *n* = 95. (D) Wild type, *n* = 48. *Ppa-ser-5 (cbh11)*, *n* = 48 for M9 buffer, *n* = 46 for serotonin solution. *Ppa-ser-5 (cbh23)*, *n* = 48. (E) Wild type, *n* = 95. *Ppa-ser-1 (cbh3); Ppa-ser-7 (cbh1)*, *n* = 96. (F) Wild type, *n* = 48. *Ppa-ser-4 (cbh12)*, *n* = 48 for M9 buffer, *n* = 47 for serotonin solution. (G) Wild type, *n* = 48. *Ppa-mod-1 (cbh5)*, *n* = 48 for M9 buffer, *n* = 45 for serotonin solution. − serotonin and + serotonin represent M9 buffer and serotonin solution, respectively. Error bars represent the SEM. Two-way ANOVA with Tukey’ s multiple comparison tests. n.s., not significant. *P < 0.05. **P < 0.01. ***P < 0.001. ****P < 0.0001. (H) Propotions of moving versus paralyzed animals in M9 buffer and serotonin solution in wild type and *Ppa-ser-4 (cbh12)* animals. All conditions, *n* = 75. Fischer’ s exact tests. n.s., not significant. ****P < 0.0001. (I) Number of body bending within 10 s in M9 buffer in wild type and *Ppa-ser-4 (cbh12)* animals. Wild type, *n* = 29. *Ppa-ser-4(cbh12)*, *n* = 30. Error bars represent SEM. Student’s *t*-test. ***P < 0.001.

To examine the functions of serotonin receptors, we generated serotonin receptor mutants using the CRISPR/Cas9 system. We obtained mutants with insertion or deletion mutations in the coding regions, resulting in premature stop codons (Table 1; Supplementary Figure S3). We observed that the *Ppa-ser-1* mutants carried more eggs in the uterus than the wild type (Figure 2A), which is consistent with a previous report that egg-laying is one of the serotonin-related behaviors in *P. pacificus* (Okumura *et al.* 2017). We also examined the number of eggs laid over 2 h in M9 buffer with or without serotonin. In the wild type, the number of eggs laid in M9 buffer containing serotonin was slightly decreased compared with that in normal M9 buffer (Figure 2B), although previous studies showed that the number of eggs laid in serotonin solution was almost half of that in M9 buffer (Gutierrez and Sommer 2007; Okumura *et al.* 2017). This difference may be caused by slight differences in experimental conditions. With the *Ppa-ser-1* mutant, the number of eggs laid in M9 buffer declined to half of that laid by the wild type, and exogenous serotonin did not affect the number of eggs in the *Ppa-ser-1* mutant (Figure 2B). This data suggests that *Ppa-ser-1* has a function to promote egg laying. Since *Ppa-ser-7* and *Ppa-ser-5* were expressed in the vulval muscles, we also examined egg-laying behavior in these mutants. We found that *Ppa-ser-7* mutants exhibited a decreased number of eggs laid in M9 buffer, and egg-laying was strongly suppressed by exogenous serotonin (Figure 2C). This finding indicates that egg-laying is enhanced via the *Ppa-ser-7* serotonin receptor. Similar to the *Ppa-ser-1* mutants, *Ppa-ser-5* mutants decreased egg-laying in M9 buffer, and the number of eggs laid was not altered by exogenous serotonin (Figure 2D). The *Ppa-ser-1*; *Ppa-ser-7* double mutant showed phenotypic features of both of the serotonin receptors; the number of eggs laid in M9 buffer was similar to the *Ppa-ser-1* mutant and exogenous serotonin further decreased the number of eggs like *Ppa-ser-7* mutants (Figure 2E). This suggest that *Ppa-ser-1* and *Ppa-ser-7* function in parallel in the modulation of egg-laying. Since previous studies showed that *Cel-ser-4* and *Cel-mod-1* have inhibitory roles in egg laying in *C. elegans* (Carnell *et al.* 2005; Hapiak *et al.* 2009), we wondered whether those serotonin receptors also suppress egg laying in *P. pacificus*. However, *Ppa-ser-4* and *Ppa-mod-1* mutants rather decreased the number of eggs laid both in M9 buffer and serotonin solution (Figure 2, F and G). These results suggest that all of the serotonin receptors play a role in egg-laying behavior. As the exogenous serotonin suppressed egg-laying greatly in the *Ppa-ser-7* mutants, we could not exclude a possibility that some of the serotonin receptors might have both stimulating and suppressive functions in egg-laying in different cells.

**Table 1:** The mutants of serotonin receptors in *P. pacificus*

Gene	Allele	Mutation	Types of mutation
***Ppa-ser-1***	*cbh3*	5 bp deletion	Frame-shift
	*cbh58*	17 bp deletion	Frame-shift
***Ppa-ser-4***	*cbh12*	10 bp deletion	Frame-shift
***Ppa-ser-5***	*cbh11*	10 bp deletion	Frame-shift
	*cbh23*	5 bp deletion	Frame-shift
***Ppa-ser-7***	*cbh1*	4 bp insertion, 1 bp SNP	Frame-shift
	*cbh2*	4 bp deletion	Frame-shift
***Ppa-mod-1***	*cbh4*	11 bp deletion	Frame-shift
	*cbh5*	13 bp deletion	Frame-shift

Because *Ppa-ser-4* was expressed in head and tail neurons whose neurites extended toward the body wall, we suspected that the *Ppa-ser-4* mutant may play a role in locomotor behavior. We examined the locomotor behavior in M9 buffer containing 30 mM of serotonin (Figure 2H). Both wild type and the *Ppa-ser-4* mutant strains were moving in M9 buffer without exogenous serotonin. While 89.3% of the wild-type animals were paralyzed by the serotonin solution, only 33.3% of *Ppa-ser-4* mutant worms were not moving in the same condition (Figure 2H). This result indicates that the *Ppa-ser-4* mutant is resistant to the paralyzing effect of serotonin, similar to *Cel-ser-4* mutants in *C. elegans* as previously reported (Gürel *et al.* 2012). Also, we investigated the body bending rate in M9 buffer without serotonin in the *Ppa-ser-4* mutant. The mutants showed body bending more frequently than wild-type animals (Figure 2I). These data suggest that *Ppa-ser-4* has an inhibitory role in locomotion in response to serotonin. Together, these serotonin receptor mutations are likely to be loss-of-function alleles and can be used for behavioral analysis (see also Table 2).

**Table 2:** Phenotypes observed in the serotonin receptor mutants

Genes	Predation	Bacterial feeding	Egg-laying	Serotonin sensitivity in locomotion
*Ppa-ser-1*	+ (defective in double mutant with *Ppa-ser-7*)	+	Defective	N/A
*Ppa-ser-4*	+	+	Defective	Negative
*Ppa-ser-5*	defective	+	Defective	N/A
*Ppa-ser-7*	+ (defective in double mutant with *Ppa-ser-1*)	Defective	Defective	N/A
*Ppa-mod-1*	+	Defective	Defective	N/A

All of the serotonin receptor mutants we generated showed some defects in behaviors. +, same phenotype as wild type. N/A, not examined.

### Predatory feeding behavior was decreased in *Ppa-ser-5* mutants and the *Ppa-ser-1*; *Ppa-ser-7* double mutant

**Figure 3:**
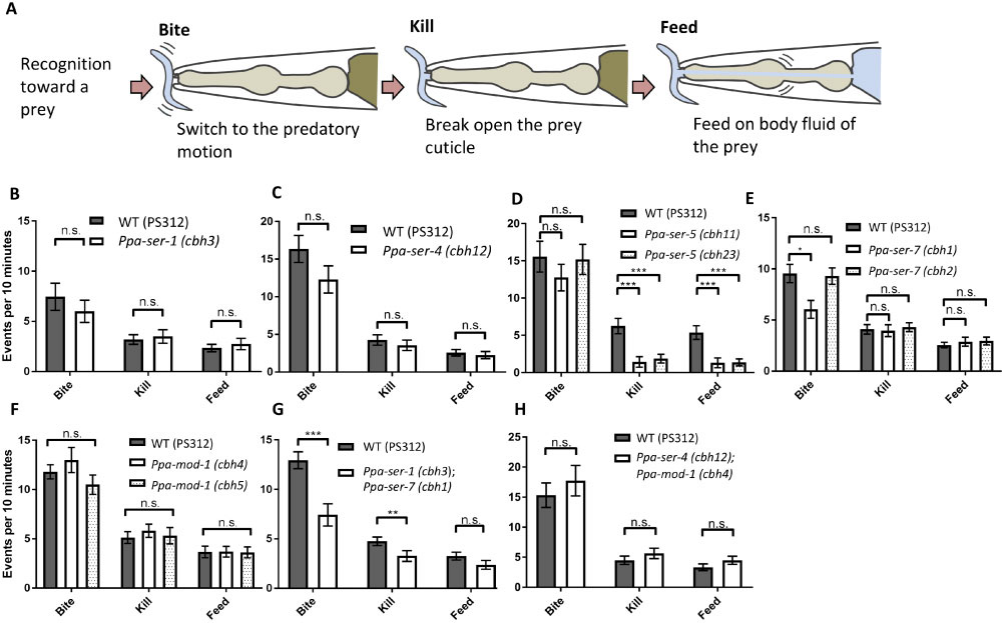
*Ppa-ser-1*, *Ppa-ser-5*, and *Ppa-ser-7* are required for predatory feeding behaviors. (A) Schematic of the three predatory events in *P. pacificus*. (B)–(I) The number of bites, kill, and feed events in 10 min in single mutants (B–F) and multiple serotonin receptor mutants (G–H). (B, C, and E), *n* = 20. (D and F), *n* = 16. (G and H), *n* = 19. WT, wild type. Error bars represent the SEM. Student's *t*-tests were performed for (B, C, G, and H). One-way ANOVA with Dunnett's multiple comparisons was used for other single mutants. n.s., not significant. *P < 0.05. **P < 0.01. ***P < 0.001.

We analyzed the predatory feeding behavior in serotonin receptor mutants using the “bite assay” to quantify predatory feeding events (Wilecki *et al.* 2015). We counted the number of the three types of predatory feeding events exhibited. Bite, kill, and feed events were characterized by the restriction of prey movement, breaking open the prey cuticle, and consuming the prey body fluid, respectively (Figure 3A). Among the five serotonin receptor mutants, *Ppa-ser-5* mutants showed decreased “kill” and “feed” events, while the number of “bite” event was not altered compared with wild-type worms (Figure 3D). This is a specific phenotype in the *Ppa-ser-5* mutants; *Ppa-tph-1* and *Ppa-bas-1* mutants showed decreased numbers of all the predatory events (Okumura *et al.* 2017). Single mutants of other serotonin receptors did not show a decrease in the number of predatory feeding events, except for one of the two alleles of *Ppa-ser-7* mutants (Figure 3, B, C, E, and F). While the *Ppa-ser-7* (*cbh1*) allele brought a stop codon immediately after the native amino acid sequence, there were 55 extra amino acids before the premature stop codon in the *Ppa-ser-7* (*cbh2*) allele (Supplementary Figure S3). This difference might cause different phenotypes between the two alleles.

In *C. elegans*, the pharyngeal pumping rate is modulated via the *Cel-ser-7* serotonin receptor and partially via the *Cel-ser-1* serotonin receptor. The *Cel-ser-7*; *Cel-ser-1* double mutant abolishes the upregulation of the bacterial feeding rate (Hobson *et al.* 2006). We hypothesized that serotonin receptors work redundantly, and the double mutant for these serotonin receptors might decrease predatory feeding behavior in *P. pacificus*. The *Ppa-ser-1*; *Ppa-ser-7* double mutant decreased predatory feeding events, especially the bite event (Figure 3G). The *Ppa-ser-4*; *Ppa-mod-1* double mutant did not show significant changes in the number of predatory events (Figure 3H), implying that these serotonin receptors are not required for the regulation of predatory feeding behavior. These data suggest that *Ppa-ser-5* is important for prey killing and *Ppa-ser-1* and *Ppa-ser-7* have redundant roles in biting and killing prey during predation.

### *Ppa-ser-1* and *Ppa-ser-7* are required for tooth movement during predatory feeding and the response to exogenous serotonin

**Figure 4:**
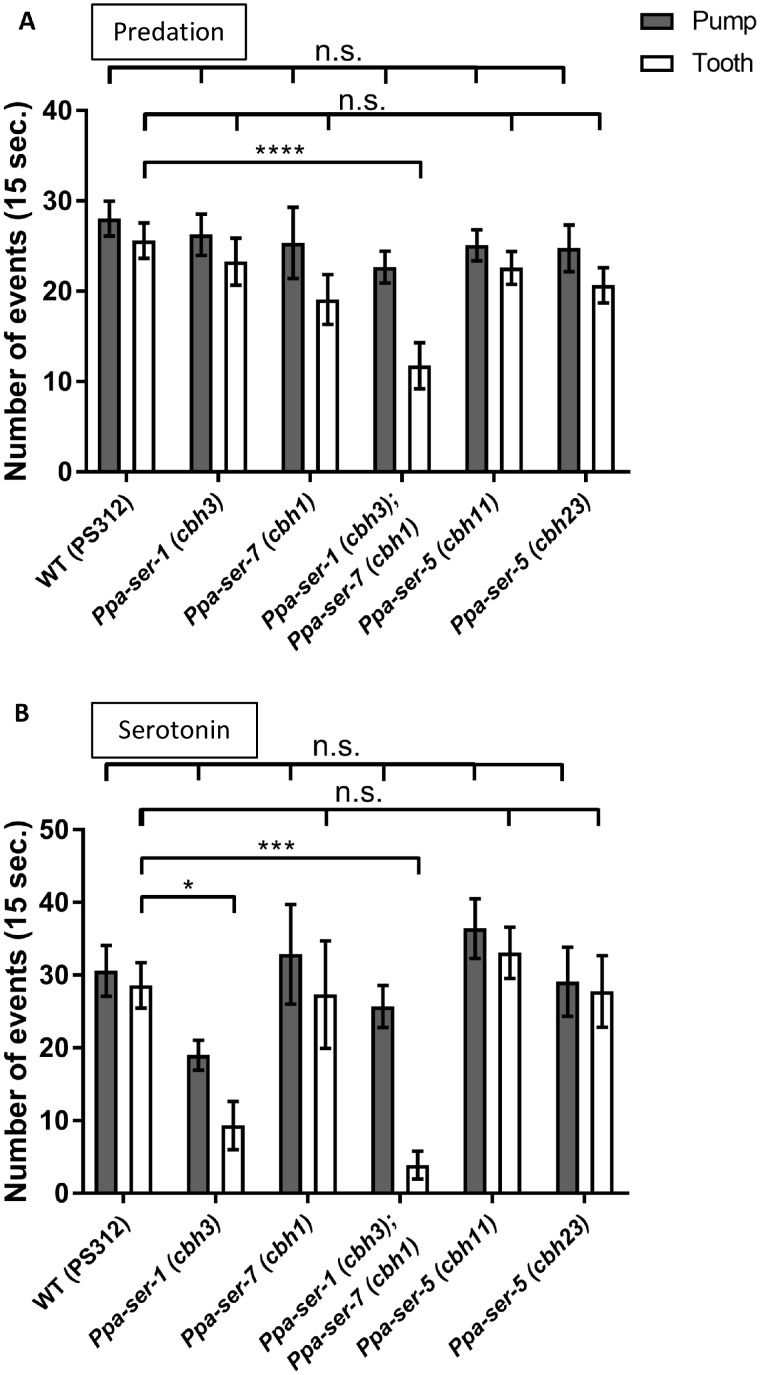
*Ppa-ser-1* and *Ppa-ser-7* work redundantly in regulating tooth movement. (A and B) The number of pharyngeal pumping and tooth movement events in 15 s in wild type (PS312), *Ppa-ser-1*, *Ppa-ser-7*, *Ppa-ser-1*; *Ppa-ser-7*, and *Ppa-ser-5* strains during predation (A) and in response to exogenous serotonin (B). Black and white bars represent pumping and tooth movement, respectively. (A) Wild type, *n* = 16. *Ppa-ser-1*, *n* = 11. *Ppa-ser-7*, *n* = 11. *Ppa-ser-1; Ppa-ser-7*, *n* = 13. *Ppa-ser-5 (cbh11)*, *n* = 9. *Ppa-ser-5 (cbh23)*, *n* = 9. (B) Wild type, *n* = 10. *Ppa-ser-1*, *n* = 10. *Ppa-ser-7*, *n* = 9. *Ppa-ser-1; Ppa-ser-7*, *n* = 10. *Ppa-ser-5 (cbh11)*, *n* = 12. *Ppa-ser-5 (cbh23)*, *n* = 9. WT, wild type. Error bars represent the SEM. One-way ANOVA with Dunnett’s multiple comparison tests. n.s., not significant. *P < 0.05. ***P < 0.001. ****P < 0.0001.

To evaluate the role of serotonin receptors in predatory feeding events, we counted pharyngeal pumping and tooth movement events during predation in *Ppa-ser-5*, *Ppa-ser-1*, and *Ppa-ser-7* mutants (Figure 4A). During predation, wild-type animals showed almost the same rate of pharyngeal pumping and tooth movement as previously described (Wilecki *et al.* 2015). Strikingly, the *Ppa-ser-1*; *Ppa-ser-7* double mutant showed a significant decrease in tooth movements, whereas the single mutants did not. Likewise, tooth movements in response to exogenous serotonin were much lower in the *Ppa-ser-1*; *Ppa-ser-7* double mutant compared with wild-type worms (Figure 4B). In this context, the *Ppa-ser-1* mutant also exhibited decreased tooth movements, while the reduction was weaker than that observed with the *Ppa-ser-1*; *Ppa-ser-7* double mutant. Unexpectedly, the *Ppa-ser-5* mutants, which showed reduced kill and feed events in the bite assay, did not show any reduction in the number of pharyngeal pumping and tooth movements during predatory feeding or following exposure to serotonin (Figure 4). These results suggest that *Ppa-ser-1* and *Ppa-ser-7* redundantly enhance tooth movement in response to serotonin, while *Ppa-ser-5* is not related to tooth movement or feeding rhythms.

### *Ppa-ser-7* and *Ppa-mod-1* mutants exhibit decreased pharyngeal pumping during bacterial feeding

**Figure 5:**
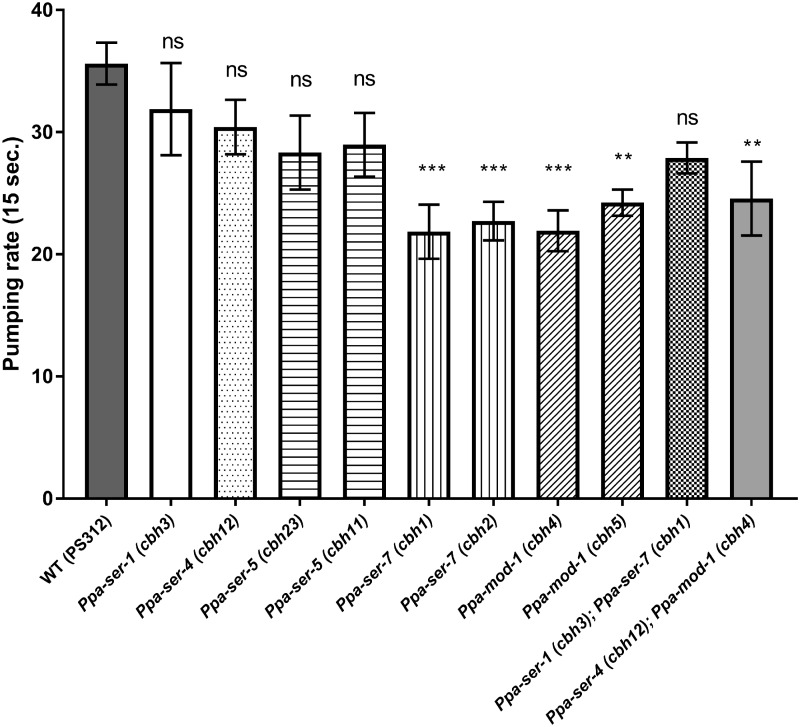
*Ppa-ser-7* and *Ppa-mod-1* play a major role in bacterial feeding. The number of pharyngeal pumping events during feeding on *E. coli* OP50 in 15 s in single and multiple serotonin receptor mutants. Wild type, *n* = 20. *Ppa-ser-1*, *n* = 10. *Ppa-ser-4*, *n* = 15. *Ppa-ser-5 (cbh11)*, *n* = 15. *Ppa-ser-5 (cbh23)*, *n* = 10. *Ppa-ser-7*, *n* = 10. *Ppa-mod-1*, *n* = 10. *Ppa-ser-1; Ppa-ser-7*, *n* = 10. *Ppa-ser-4; Ppa-mod-1*, *n* = 10. WT, wild type. Error bars represent the SEM. One-way ANOVA with Dunnett’s multiple comparisons. n.s., not significant. **P < 0.01. ***P < 0.001.

Serotonin also contributes to the upregulation of pharyngeal pumping during bacterial feeding in both *P. pacificus* and *C. elegans* (Sze *et al.* 2000; Okumura *et al.* 2017; Ishita *et al.* 2020). We investigated whether the downstream pathway regulating pumping during bacterial feeding is conserved between these species. We examined the pharyngeal pumping rate in serotonin receptor mutants during bacterial feeding (Figure 5). Single mutants of *Ppa-ser-7* and *Ppa-mod-1*, and the *Ppa-ser-4*; *Ppa-mod-1* double mutant showed a significant decrease in the pharyngeal pumping rate. Other single mutants and multiple mutants of serotonin receptors did not show any alteration in the pumping rate during bacterial feeding. Curiously, the *Ppa-ser-1*; *Ppa-ser-7* double mutant did not decrease the pumping rate on OP50, even though it carried mutation in *Ppa-ser-7*. The *Ppa-ser-1* mutation may mask the effects of the *Ppa-ser-7* mutation. Together, these data suggest that *Ppa-ser-7* and *Ppa-mod-1* play a major role in the upregulation of the pumping rate during bacterial feeding in *P. pacificus*.

## Discussion

**Figure 6:**
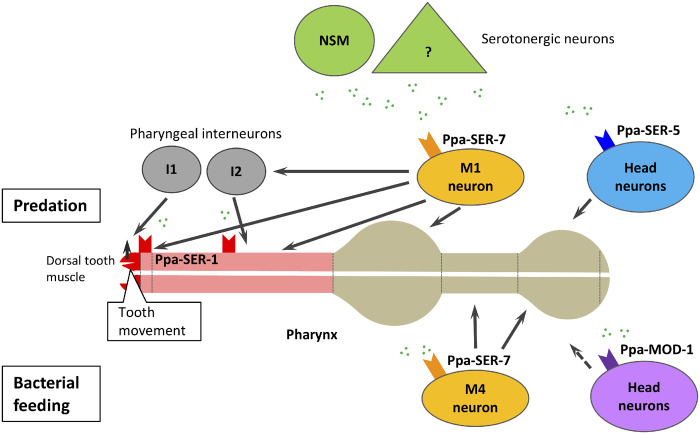
A hypothetical model for serotonergic modulation of predatory and bacterial feeding. For the regulation of predation, serotonin released from serotonergic neurons (shown in green) activates the M1 neuron via the *Ppa-ser-7* serotonin receptor. The M1 neuron innervating dorsal tooth muscle cells transmits neuronal signals to induce tooth movement. The M1 neuron may also activate I1 and I2 neurons, resulting in indirect stimulation of the anterior pharyngeal muscles. In parallel, the anterior pharyngeal muscles (shown in pink), including the dorsal tooth muscles (shown in red), can be activated by serotonin directly via Ppa-SER-1. These neural pathways may work redundantly; thus, only the *Ppa-ser-1*; *Ppa-ser-7* double mutant showed tooth movement defects. While the functional roles of *Ppa-ser-5* are not clear, it may function in some head neurons to promote the efficient killing of prey. For the modulation of bacterial feeding, serotonin activates the M4 pharyngeal neuron via Ppa-SER-7, resulting in stimulating posterior parts of the pharynx, promoting peristalsis of the posterior pharynx and partially pharyngeal pumping. Ppa-MOD-1 may have a role in the head neurons and indirectly upregulate pharyngeal pumping, perhaps via weakening the activation of feeding inhibitory neurons.

Although serotonin regulates feeding behaviors, the downstream neuronal mechanisms have not been elucidated previously in *P. pacificus*. In this study, we demonstrated the functions of *Ppa-ser-1*, *Ppa-ser-5*, and *Ppa-ser-7* in predatory feeding behavior and *Ppa-ser-7* and *Ppa-mod-1* in bacterial feeding behavior by utilizing CRISPR/Cas9 knockout mutants. We also identified cells expressing serotonin receptors. These cells are candidates for the target of serotonin in modulating feeding behaviors in this animal. This study suggests putative downstream mechanisms of serotonergic modulation in predatory and bacterial feeding behaviors (Figure 6).

In this study, we showed that two serotonin receptors, *Ppa-ser-1* and *Ppa-ser-7*, redundantly induce tooth movement during predation and in response to exogenous serotonin. *Cel-ser-1* is expressed throughout the pharyngeal muscles of *C. elegans* (Tsalik *et al.* 2003; Carnell *et al.* 2005), while *Ppa-ser-1* is expressed only in the anterior parts in *P. pacificus* (Figure 1B). In addition, the expression of the *Ppa-ser-1* RFP reporter was prominent in the dorsal tooth muscle (pm1) compared with the other pharyngeal muscles (Figure 1B). This finding suggests that the alteration of expression patterns of serotonin receptors may play a role in the evolutional acquisition of predatory feeding behavior. Furthermore, a molluskan study showed a correlation between the expression of serotonin receptors in a homologous neuron and species-specific swimming behavior, implying that the expression of serotonin receptors in specific cells may be important for the presence of a specific behavior (Tamvacakis *et al.* 2018). Supporting this idea, the *Ppa-ser-1* mutant showed the greatest defect in tooth movements in response to exogenous serotonin among single serotonin receptor mutants. We should note that the promoter fragments used in this study might overlap with the potential regulatory regions of adjacent genes. To verify *bona fide* expression patterns, evaluation of the expression patterns using knock-in of tag sequences in genomic loci or other methods such as single-molecule fluorescence *in situ* hybridization (smFISH) might be useful in future studies.

*Ppa-ser-7* was expressed in the pharyngeal neurons (Figure 1E), whose expression patterns were different from those of *Ppa-ser-1*. Among *Ppa-ser-7*-expressing neurons, the M1 pharyngeal neuron is the most probable candidate for the regulation of tooth movement (Figure 6). There are four empirical reasons for this hypothesis. First, the M1 neuron has synaptic connectivity with the anterior parts of the pharynx, including pm1 and g1D gland cells that may be involved in predation (Bumbarger *et al.* 2013; Riebesell and Sommer 2017). Second, a previous *C. elegans* study revealed that the M1 neuron is responsible for “spitting” behavior, which opens the anterior tip of the pharynx and expels the contents of the pharynx, implying that the M1 neuron is capable of stimulating anterior tip muscles (Bhatla *et al.* 2015). Third, the M1 neuron also has synaptic connectivity with I1 and I2 neurons, which are also presynaptic to the anterior pharyngeal muscles. These connections are not seen in *C. elegans*, implying that the functions of these neurons change to regulate the predatory feeding in *P. pacificus* (Bumbarger *et al.* 2013). Finally, *Cel-ser-7* is not expressed in the M1 neuron in *C. elegans* (Hobson *et al.* 2006). Therefore, the M1 pharyngeal neuron may have direct or indirect roles in stimulating the dorsal tooth muscles, and serotonin modulates the tooth movement via the M1 neuron. To prove this model, cell-ablation or neuron-specific rescue experiments for those neurons should be conducted in future studies. Conversely, the M4 neuron, another *Ppa-ser-7* expressing cell, was previously described as a neuron regulating peristalsis in the posterior pharyngeal muscles and partial pharyngeal pumping (Chiang *et al.* 2006), suggesting a role for serotonin in the peristalsis of the posterior pharynx. In *C. elegans*, the M4 neuron modulates isthmus peristalsis via Cel-SER-7 (Song and Avery 2012), suggesting that the role of SER-7 in the M4 neuron might be conserved between the two species.

We also revealed that *Ppa-ser-5* decreased the number of “kill” and “feed” events, but not “bite” event during predatory feeding behavior (Figure 3D). This feature is not observed in serotonin synthesis mutants or other serotonin receptor mutants (Okumura *et al.* 2017 and Figure 3, B, C, E, and F). In addition, *Ppa-ser-5* mutants did not decrease the number of pharyngeal pumping and tooth movements during predatory feeding behavior or in response to serotonin (Figure 4). Interestingly, *Ppa-ser-5* was expressed in some sensory neurons, including amphid neurons and labial neurons (Figure 1D). If *Ppa-ser-5* is involved in predation via these sensory neurons, serotonin might modulate the activity of sensory neurons during prey-sensing. Serotonergic modulation of the activity of sensory neurons has been reported in *C. elegans* studies. The pair of ASH amphid neurons are activated by nose touch only when the amount of serotonin is abundant (Hilliard *et al.* 2005), and it seems to be modulated via Cel-SER-5 (Harris *et al.* 2009). Although we could not specify the amphid neurons that express *Ppa-ser-5*, similar activation mechanisms might be related to prey sensing and the subsequent predatory feeding behavior in *P. pacificus*.

Consistent with the studies in *C. elegans*, *P. pacificus* also requires serotonin for fast pharyngeal pumping during bacterial feeding (Okumura *et al.* 2017). We showed that single mutants of *Ppa-ser-7* and *Ppa-mod-1* failed to increase pharyngeal pumping during bacterial feeding (Figure 5). These results are different from the results of *C. elegans* studies in three aspects. First, the *P. pacificus Ppa-ser-7* mutants exhibited a lower pumping rate during bacterial feeding on agar plates. While a study using the HB101 strain as a bacterial food showed similar results for *C. elegans* (Song and Avery 2012), most of the *C. elegans* studies showed that the single *Cel-ser-7* mutations do not decrease the bacterial feeding rate. Second, in *P. pacificus, Ppa-mod-1* mutants decreased the pumping rate during bacterial feeding. This defect has not been reported in *C. elegans Cel-mod-1* mutants, even in a study that revealed that *Cel-mod-1* functions in the upregulation of pharyngeal pumping by suppressing feeding downregulation circuits (Liu *et al.* 2019). Third, while the *Cel-ser-1*; *Cel-ser-7* double mutant significantly decreased the bacterial feeding rate in *C. elegans* (Hobson *et al.* 2006), the pumping rate on bacterial food in the *Ppa-ser-1*; *Ppa-ser-7* mutant was not significantly different from that of the wild type. This result is unexpected because a single mutation of *Ppa-ser-7* significantly decreased the bacterial feeding rate, implicating the possibility that the mutation in *Ppa-ser-1* blocks the effects of *Ppa-ser-7* mutation in an unknown manner. These differences between the two species may reflect the alteration of the functions of serotonin receptors during the acquisition of predatory feeding behavior; *Ppa-ser-1* shifted to specialize in regulating predatory feeding behavior, and the functions of *Ppa-ser-7* and *Ppa-mod-1* in bacterial feedings became more pronounced. To confirm this working hypothesis, functional and expressional analysis in evolutionarily related species should be performed in future studies.

This study demonstrated that different types of feeding behaviors in *P. pacificus* are under the control of distinct combinations of serotonin receptors. One of the remaining questions is how to switch bacterial feeding movements into predatory feeding movements. The switching mechanism is likely to require other neural regulatory systems; thus, studies with mutations of other neurotransmitters and neuropeptides are necessary. This switching may also be dependent on the sensory input received from the food source. While *Ppa-self-1*, a small peptide required to avoid cannibalism, has been recently identified (Lightfoot *et al.* 2019), the neuronal mechanisms of food recognition are largely unknown in *P. pacificus*. Future neurogenetic studies of food sensing will reveal how food stimuli are converted to behavioral output via the serotonergic nervous system. Another question is how the polyphenism of feeding behaviors, depending on the two mouth forms, is regulated in *P. pacificus*. We could not find marked differences in the expression patterns of serotonin receptors between predatory eurystomatous and nonpredatory stenostomatous morphs. Exploration of neuronal activity, synaptic connectivity, or transcriptional profiles might enable us to determine the differences in the nervous system between these morphs to understand the behavioral polyphenism in *P. pacificus*.
